# Myxofibrosarcomas Have Extremely Low Internal Echoes: A Case Report

**DOI:** 10.7759/cureus.102606

**Published:** 2026-01-29

**Authors:** Taiji Tamori, Shoji Oura

**Affiliations:** 1 Department of Surgery, Kishiwada Tokushukai Hospital, Kishiwada, JPN

**Keywords:** extremely low internal echoes, high signals on t1-weighted images, low suv max values, mucus, myxofibrosarcoma

## Abstract

We report a rare case of myxofibrosarcoma characterized by extremely low internal echoes on ultrasonography, a feature that may serve as an important diagnostic clue. The patient was a 54-year-old woman with a medical history of treatment for primary breast cancer as well as axillary recurrence treatment. After a prolonged disease-free interval of 15 years, the patient detected an ipsilateral axillary mass and was referred to our hospital. Ultrasound revealed a well-circumscribed axillary mass, measuring 21 mm, with extremely low internal echoes. MRI of the axillary mass showed faintly high signals on fat-suppressed T1-weighted images, predominantly high signals on T2-weighted images, and persistent enhancement accompanied by small non-enhanced areas on dynamic studies. Positron emission tomography (PET) showed a maximal standardized uptake value (SUVmax) of 3.1. With a working diagnosis of either axillary re-recurrence or a form of axillary sarcoma, we successfully resected the axillary mass for both diagnostic confirmation and potential curative intent. Postoperative pathological evaluation demonstrated atypical stellate cells with abundant chromatin growing in sparse and focally dense patterns against a mucus-rich background. Immunostaining showed AE1/AE3 negativity and a Ki-67 labeling index of 40%, leading to a final diagnosis of myxofibrosarcoma. Given the patient’s preferences and prior exposure to chemotherapy and radiotherapy for breast cancer, she did not receive any adjuvant therapy and has been followed without recurrence on an outpatient basis for 11 months. Diagnostic physicians should be aware that myxofibrosarcomas may exhibit extremely low internal echoes, weak high signals on fat-suppressed T1-weighted images, and low SUVmax values on PET, findings that are attributable to the mucus-rich background.

## Introduction

Soft tissue malignant tumors, such as liposarcoma, occur much less frequently than epithelial solid malignancies, including lung cancer and breast cancer [1]. Myxofibrosarcoma, a subtype of rare malignant soft tissue tumors, was formerly called malignant fibrous histiocytoma (MFH) and has traditionally been managed primarily with surgical treatment [2-4]. Conversely, oncologists have long struggled to treat myxofibrosarcoma using systemic approaches due to the lack of well-established and effective regimens. It is well known that myxofibrosarcomas occur most frequently in the lower extremities, but they may also arise in relatively superficial locations throughout the body outside the lower extremities [1,4]. In addition to CT and MRI, ultrasound may play a valuable role in the diagnosis of the exceedingly rare occurrence of myxofibrosarcoma arising in the breasts and axillae [5]. A very limited number of studies have reported imaging characteristics of myxofibrosarcoma in comparison with other non-epithelial disorders, especially on MRI [6,7]. Few studies, however, have reported the ultrasonographic features of myxofibrosarcoma to date.

Many breast surgeons have come across axillary recurrence after sentinel lymph node biopsy for breast cancer, but rarely encounter recurrence after axillary dissection [8]. On the other hand, physicians cannot speculate on what kind of tumors arise in the dissected axilla due to the extremely low incidence. The vast majority of physicians, therefore, naturally suspect lymph node recurrence even after axillary dissection when new, well-circumscribed masses appear in the dissected axilla. We report the case of a breast cancer patient who developed myxofibrosarcoma in the dissected axilla, and describe the imaging characteristics of myxofibrosarcoma through an evaluation of pathological and imaging findings.

## Case presentation

**Figure 1:**
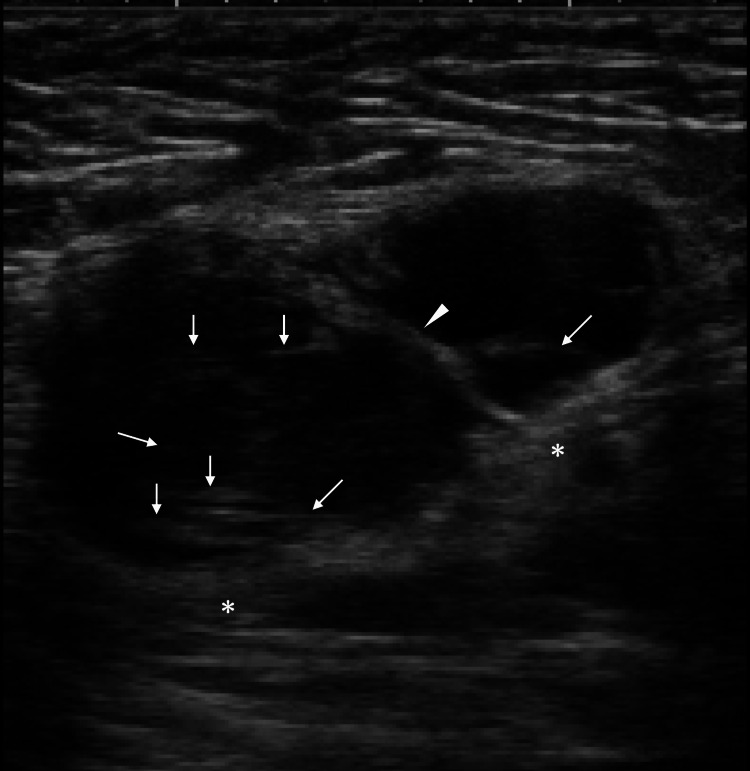
Ultrasound findings Ultrasound showed a lobulated mass deep in the axilla with 21×19×15 mm in size, distinct margins, slight peri-tumoral vascularity, very low internal echoes, enhanced posterior echoes (asterisk), an intra-tumoral septum (arrowhead), and scant punctate and linear high echoes (arrows)

The patient was a 54-year-old woman who had previously undergone breast-conserving surgery and sentinel biopsy without adjuvant radiotherapy for right breast cancer 18 years earlier. The patient had developed an axillary recurrence and undergone salvage axillary dissection 15 years earlier. At that time, she had received adjuvant anti-human epidermal growth factor receptor type 2 (HER2) agent-containing chemotherapy and radiotherapy to the conserved breast, the supraclavicular region, and the chest wall. After a very long 15-year disease-free interval, the patient noticed an axillary mass and visited our hospital for a detailed examination. Ultrasound showed a lobulated mass with distinct margins, extremely low internal echoes, focal punctate or linear high echoes, a presumed septum, and enhanced posterior echoes (Figure 1).

**Figure 2:**
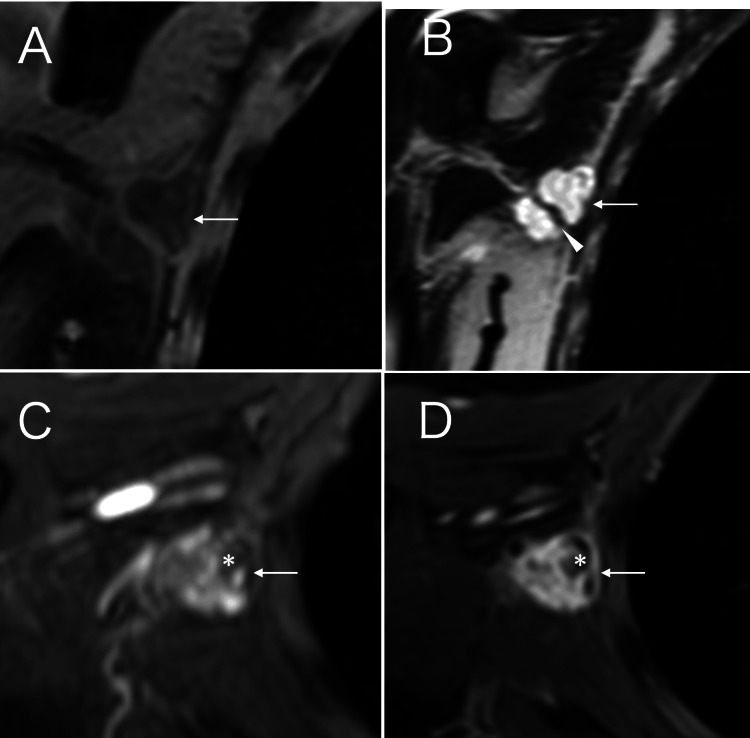
MRI findings A. MRI of the tumor (arrow) showed slightly high signals on fat-suppressed T1-weighted images. B. MRI of the tumor (arrow) showed strong high signals with linear low signals corresponding to the intra-tumoral septum (arrowhead) on T2-weighted images. C. MRI of the tumor (arrow) showed enhancement with focal non-enhanced areas (asterisk) on early phase dynamic studies. D. MRI of the tumor (arrow) showed stronger enhancement with retained no enhanced areas (asterisk) on late phase dynamic studies MRI: magnetic resonance imaging

MRI of the axillary mass showed weak high signals on fat-suppressed T1-weighted images, high signals on T2-weighted images, and retained enhancement showing a persistent pattern with small no enhancement areas on dynamic studies (Figure 2).

**Figure 3:**
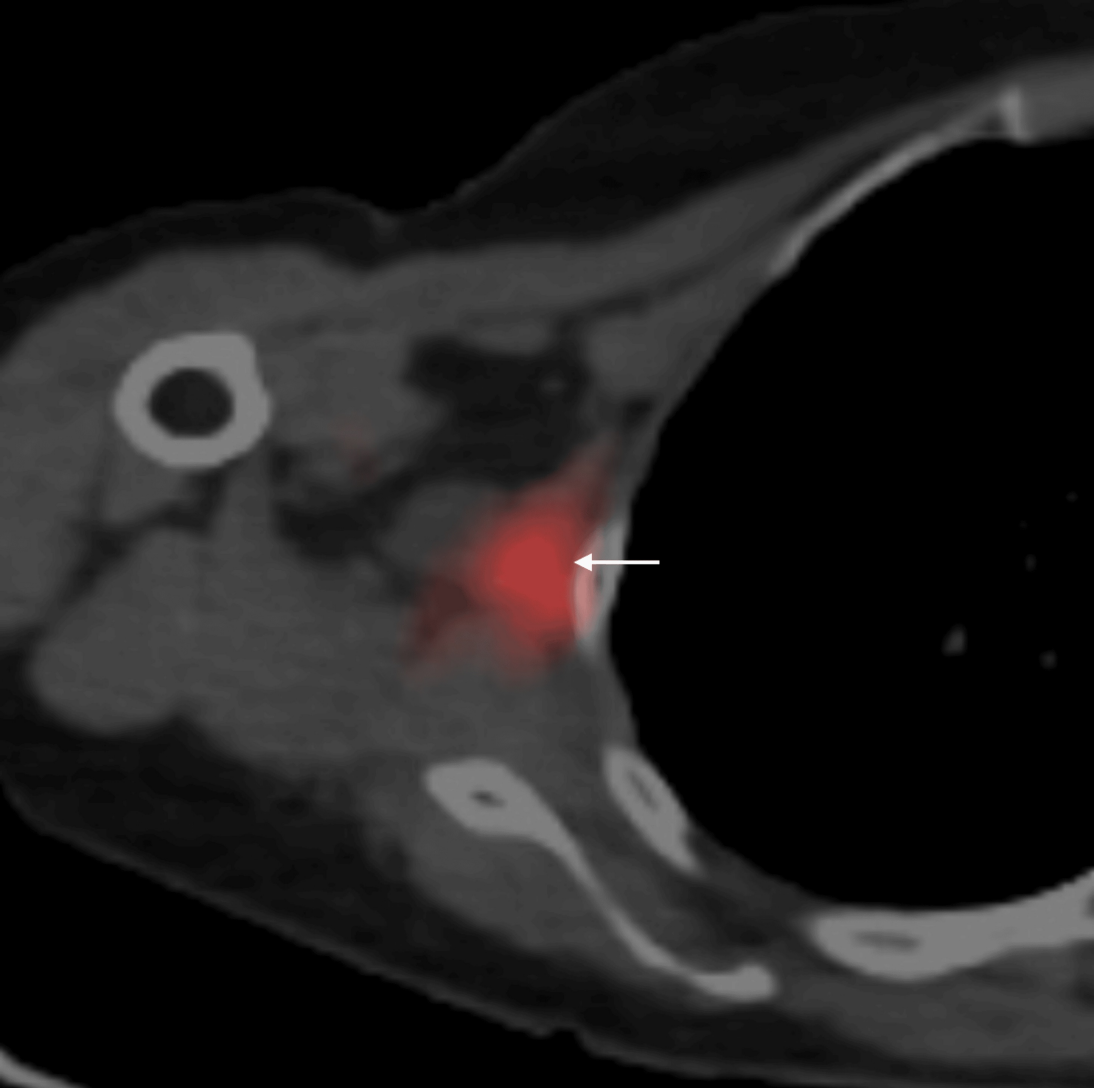
PET/CT findings PET/CT of the target mass showed a maximal standardized uptake value of 3.1 (arrow). Details of the PET examination were as follows: body weight: 59.6 kg, blood sugar: 85 mg/dL, fluorodeoxyglucose (FDG) dose: 182.6MBq, start of PET examination: 60 minutes after FDG administration, scanner protocol 1 view/2 minutes PET/CT: positron emission tomography/computed tomography

Positron emission tomography/CT (PET/CT) using fluorodeoxyglucose showed a maximal standard uptake value (SUVmax) of 3.1 in the target mass but no avid uptake other than the axilla (Figure 3).

**Figure 4:**
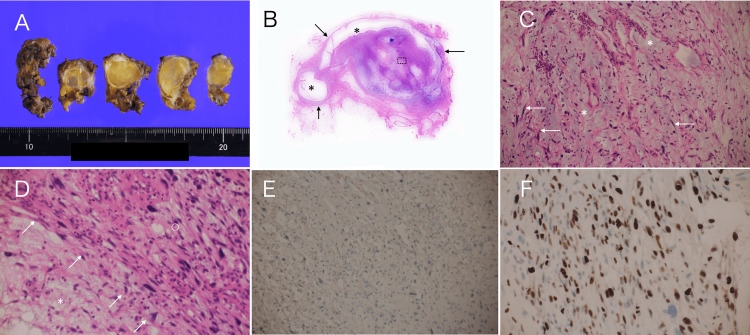
Pathological findings A. Cut surfaces of the resected tumor showed complete resection of the tumor. B. Low magnified view showed no interruption of the tumor capsule (arrows) and acellular areas (asterisks). C. Magnified view showed stellate cells (arrows) against the mucus-rich background (asterisks). D. Magnified view of the dotted square in Figure 4B showed hypo cellular areas (asterisk) and hyper cellular areas (open circle). Interfaces between the two areas (arrows) presumably generated linear high echoes. E. Immunostaining showed AE1/AE3 negativity. F. Immunostaining showed a Ki-67 labelling index of 40%

These imaging findings and the difficulty in excluding other disorders made us speculate that the target mass was axillary lymph node re-recurrence. Therefore, under the tentative diagnosis of surgically curable lymph node recurrence, we performed resection of the axillary mass and achieved complete resection. Postoperative pathological examination showed atypical stellate cells with abundant chromatin growing in sparse and focally dense patterns against a mucus-rich background, with no apparent mitosis. Immunostaining showed AE1/AE3 negativity and a Ki-67 labeling index of 40%, leading to the diagnosis of myxofibrosarcoma (Figure 4).

In light of the patient’s preferences, prior exposure to chemotherapy and radiotherapy for recurrent breast cancer, and the absence of established treatment regimens for myxofibrosarcoma, we decided to follow the patient without applying either chemotherapy or radiotherapy. The patient has been well for 11 months on an outpatient basis and is scheduled for long-term follow-up.

## Discussion

Myxofibrosarcomas generally have an abundant background of mucus [6,7]. Diagnostic physicians, therefore, should fully take into account the effect of mucus on imaging findings when diagnosing suspected myxofibrosarcomas. The presence of abundant mucus generally generates extremely low internal echoes, slightly high signals on T1-weighted images, and strongly high signals on T2-weighted images. In addition, PET findings, which can not only show tumor morphology but also tumor biology, can be markedly affected by the presence of mucus [9]. In short, abundant mucus resulted in lower SUVmax values, namely 3.1, despite the high Ki-67 labeling index of 40% in this case [10].

Weakly high signals on fat-suppressed T1-weighted images strongly suggest the presence of mucus in the target tumor, which was confirmed by a postoperative pathological study [11]. In addition, strong high signals on T2-weighted images reflected the abundant protons in the mucus. Almost all inflammation and tumors are essentially at least somewhat watery, which overwhelmingly causes them to show low signals on T1-weighted images. Some facilities, therefore, do not acquire T1-weighted images when performing MRI examinations of target tumors. MRI findings in this case clearly showed the usefulness of T1-weighted images for estimating the pathological components that make up the tumor when interpreting MRI images of suspected malignancies. Diagnostic physicians, therefore, should note that T1-weighted and fat-suppressed sequences may help characterize the myxoid content.

Ultrasound showed that the axillary mass had extremely low internal echoes in this case. It is well known that differences in acoustic impedance among the pathological components constituting a tumor determine the internal echoes [12]. In short, substances with markedly different acoustic impedance, such as adipocytes and calcifications, when present within a tumor, generate internal high echoes due to ultrasound wave backscattering. Conversely, substances with similar acoustic impedance can generate very low internal echoes, as seen in malignant lymphomas and medullary carcinomas of the breast [13,14]. In addition to the extremely low echoes, atypical stellate cells and interfaces between sparse and dense cell areas in the background mucus generated punctate and linear high echoes in this case. In short, ultrasound wave backscattering or reflection occurs at the scattering bodies, namely tumor cells, and at the interfaces between cell-rich and cell-sparse areas, which led to the formation of punctate or linear high echoes, respectively. In addition, the absence of intratumoral fibrous components resulted in enhanced posterior echoes in this case.

This disorder naturally requires differential diagnosis from cystic lesions, necrotic lymph node metastasis, and myxoid liposarcoma. A fat-suppressed T1-weighted image can easily lead to the exclusion of the first two diseases. Lastly, diagnostic physicians should also note that, depending on pathological findings, some myxoid liposarcomas may have very similar imaging findings to those of myxofibrosarcoma. Although many studies have reported the therapeutic efficacy of treatments for myxofibrosarcoma, limited studies have evaluated the imaging findings of these tumors to date. This report suggests that myxofibrosarcomas may show very low internal echoes, enhanced posterior echoes, slightly high signals on T1-weighted images, and relatively low SUVmax values.

## Conclusions

Myxofibrosarcomas may have low SUVmax values on PET and weakly high signals on T1-weighted MRI, both due to the presence of background mucus. Myxofibrosarcomas may further have very low internal echoes and enhanced posterior echoes due to the absence of adipocytes and fibrous components. It, therefore, is very important for diagnostic physicians to keep myxofibrosarcomas in mind when diagnosing well-circumscribed solid tumors with these imaging findings near the body surface.
